# A national survey of publicly funded chronic pain management services in Ireland

**DOI:** 10.1007/s11845-021-02673-5

**Published:** 2021-06-10

**Authors:** Andrew Purcell, Keshava Channappa, David Moore, Dominic Harmon

**Affiliations:** 1 Department of Anaesthesiology and Pain Medicine, St. Vincent’s University Hospital, Dublin, Ireland; 2 Department of Anaesthesiology and Pain Medicine, Beaumont Hospital, Dublin, Ireland; 3 Department of Anaesthesiology and Pain Medicine, University Hospital Limerick, Limerick, Ireland

**Keywords:** Chronic pain, Ireland, Pain management, Services, Survey

## Abstract

**Background:**

Chronic pain management services have historically been under-resourced in Ireland. There is no agreed model of care for chronic pain management services in Ireland. Previous studies have assessed the extent of services in Ireland without examining waiting times for access to services.

**Aims:**

This study aimed to quantify the extent of, geographical distribution of and waiting times for access to publicly funded chronic pain management services in Ireland.

**Methods:**

Using the British Pain Society’s Core Standards for Pain Management Services in the UK (2015) and International Association for the Study of Pain (IASP) recommendations, a questionnaire was devised. Publically funded departments in Ireland were contacted and questionnaires completed. Waiting list data was publicly available and obtained from the National Treatment Purchase Fund website.

**Results:**

There was a 100% response rate. Sixteen publicly funded chronic pain management services were identified. There are 27 chronic pain management consultants (16.6 whole time equivalents (WTE)) practicing chronic pain management, amounting to 0.55 specialists (0.34 WTEs)/100,000 of the population. There are 21 WTE for non-consultant hospital doctors (NCHDs), 26.5 WTEs for nursing, 8 WTEs for physiotherapy and 6.2 WTEs for psychology, nationally. A percentage of 93.75% of departments (*n* = 15) provide interventional therapies, 37.5% (*n* = 6) provide advanced neuromodulation and 43.75% (*n* = 7) are managing intrathecal pump therapies. There are five pain management programmes nationally. As of January 2020, ~ 25% patients on waiting lists for outpatient appointments were waiting > 18 months, with ~ 17% patients on waiting lists for interventional treatments waiting > 12 months.

**Conclusions:**

Shortage of multidisciplinary staff is of particular concern for Irish services. Patient access is limited as evidenced by significant waiting lists. In order to improve access to care and bring services in line with international recommendations, increased resources are needed.

## Introduction

Chronic pain is estimated to affect between 13 and 36% of the Irish population [1, 2]. Despite its prevalence and impact on patients’ quality of life, there is no national strategy for the management of chronic pain in Ireland despite a previous call for one [3].

The International Association for the Study of Pain (IASP) provides guidance on standards of care that include the approach, infrastructure and treatment content of such services, and recommended waiting times [4]. In 2015, the British Pain Society (BPS) published a document outlining the core standards for pain management services in the UK. The principle driving these standards was to have an acceptable level of care in pain management that was consistent, both geographically and from initial to escalating levels of care [5]. What followed was a series of national audits that sought to paint a clear picture of the extent and delivery of chronic pain management services in the UK [4].

There is currently no official model of care for the delivery of chronic pain management services in Ireland. While details of Irish chronic pain management services have been published before [3, 6], we sought to conduct a comprehensive follow-up taking the IASP and BPS publications into consideration from an Irish perspective, while also examining waiting times for access to said services.

## Methods

The study had full approval from the Research and Ethics Committee, University Hospital Limerick (Ref. No: 145/19).

Ireland (also referred to as the Republic of Ireland) operates a government-funded, public healthcare system that is administered by a state-run organisation known as the Health Service Executive (HSE). Organisationally individual hospitals operate as part of a hospital group system of which there are seven nationally: University of Limerick (UL) Hospitals Group, South/South-West Hospitals Group, Saolta University Health Care Group, Ireland East Hospitals Group, Dublin Midlands Hospitals Group, Royal College of Surgeons in Ireland (RCSI) Hospitals Group and Children’s Health Ireland (CHI). Healthcare in the six counties of Northern Ireland is administered by the National Health Service (NHS) of the UK and as such is not administered by the government of Ireland.

Using the standards set out in the BPS’s Core Standards for Pain Management Services in the UK (2015) document [5] and IASP recommendations for pain management services [7], we devised a 21-point questionnaire to be used to acquire relevant data about chronic pain management services in departments around Ireland. Departments in public hospitals known to be operating a chronic pain management service were surveyed. A clinician representative from each of these departments was contacted by one of the authors, and the questionnaire was completed. Departments operating in private healthcare facilities were not included in this study. Data collected included levels of staffing in medical (consultant and non-consultant hospital doctors), nursing, allied health (physiotherapy, clinical psychology, occupational therapy and clinical pharmacy) and administrative staff. Actual personnel numbers and whole time equivalents (WTEs) were recorded. Operational details including those relating to administrative facilities, outpatient clinic availability, types of treatment available, multi-disciplinary team (MDT) interaction with other medical/surgical services as well as details relating to engagement with clinical audit and recent clinical research activities were also collected by the authors. Data were collected between July and September 2020.

The national treatment purchase fund (NTPF) is a corporate body with functions and responsibilities as set out under Irish law. One of its key functions includes collecting, collating and validating information on persons waiting for public hospital treatment. Waiting list data was available in the public domain from the NTPF website.

Hospital group population data was available from individual hospital group web pages and published operational/delivery plans [8–14]. Data reported per 100,000 of population refers to the population size of the catchment area of the hospital group in question.

## Statistical analysis

Standard, descriptive statistics including frequencies, means (averages), medians and ranges were calculated using Microsoft® Excel® for Mac (Version 14.7.7).

## Results

Across the seven hospitals groups in which the HSE delivers public healthcare in Ireland, there are 16 hospitals known to operate a chronic pain management service. The authors collected the data between July and September 2020. There was a 100% response rate of self-reported data.

Nationally, there are 27 consultants delivering chronic pain management. All 27 of these practitioners have specialty training in Anaesthesiology and Pain Medicine. This represents 0.55 consultants per 100,000 of the population, delivering a total of 16.6 whole time equivalents (WTEs), equating to a national average of 0.34 WTEs per 100,000 of the population. On average, there are 2.37 WTEs per hospital group (range = 0.5–5.1; median = 2). There is wide disparity when it comes to WTE per 100,000 of the population per hospital group as is shown in Table 1.

**Table 1:** Irish chronic pain medicine services data. Data per 100,000 of the population refers to population of the hospital group. *The population served by Children’s Health Ireland is the entire paediatric population of Ireland (100%); as of 2019, this was estimated to be approximately 1.075 million^8^

Hospital groups	Ireland	University of Limerick	Saolta Health	South/south-west	Dublin Midlands	Ireland East	RCSI	Children’s Health Ireland
Academic partners	-	University of Limerick	NUI Galway	UCC	TCD	UCD	RCSI	Multiple
Percentage of population served	100	9.65	14.91	18.38	16.73	22.46	17.86	100*
Consultant staff data								
Total personnel	27	2	4	4	6	7	3	1
Total personnel/100,000	0.55	0.42	0.55	0.44	0.73	0.64	0.34	0.09
WTE/100,000	0.34	0.21	0.27	0.38	0.45	0.46	0.21	0.05
NCHD staff data								
Total personnel	22	2	2	4	5	6	2	1
Total personnel/100,000	0.45	0.42	0.27	0.44	0.61	0.55	0.23	0.09
WTE/100,000	0.43	0.21	0.27	0.44	0.61	0.55	0.23	0.09
Nursing staff data								
Total personnel	33	3	5	9	6	4	2	4
Total personnel/100,000	0.67	0.63	0.68	1	0.73	0.36	0.23	0.37
WTE/100,000	0.54	0.63	0.68	0.61	0.73	0.09	0.23	0.37
Physiotherapy staff data								
Total personnel	10	1	2	2	1	3	1	0
Total personnel/100,000	0.2	0.21	0.27	0.22	0.12	0.27	0.11	0
WTE/100,000	0.16	0.21	0.27	0.13	0.12	0.17	0.11	0
Psychology staff data								
Total personnel	8	0	2	1	1	3	1	0
Total personnel/100,000	0.16	0	0.27	0.11	0.12	0.27	0.11	0
WTE/100,000	0.13	0	0.21	0.06	0.12	0.2	0.11	0
Administrative staff data								
Total personnel	23	2	2	5	5	5	3	1
Total personnel/100,000	0.47	0.42	0.27	0.56	0.61	0.45	0.34	0.09
WTE/100,000	0.42	0.32	0.27	0.5	0.54	0.39	0.34	0.09
Advanced services data								
PMP	**-**	No	Yes	No	Yes	Yes	No	No
Neuromodulation (SCS)	**-**	No	No	Yes	Yes	Yes	Yes	No
Intra-thecal pump service	**-**	No	No	Yes	Yes	Yes	Yes	No

At the time of data collection, there were 21 non-consultant hospital doctor (NCHD) WTEs across all 16 sites (registrar and fellow grades), all of whom were NCHD anaesthesiologists. This represents, on average, 0.43 NCHD WTE per 100,000 of the population per hospital group.

There are currently 36 outpatient (OPD) chronic pain management clinics in Ireland, operating on a weekly basis (where a single OPD session is equal to a half-day clinic). On average, per hospital group, this equates to 5.14 OPD clinics per week. This equates to on average 0.6 OPD clinics per week per 100,000 of the population (range = 0.12–1.04; median = 0.55).

Only 31.25% (*n* = 5) of departments had dedicated input from medical, nursing, physiotherapy and psychology team members. 87.5% of chronic pain departments (*n* = 14) had at least specialist nursing team members in addition to medical staff.

Nursing care for chronic pain management departments is delivered by a combination of staff nurses, clinical nurse specialists (CNS) and a number of advanced nurse practitioners (ANPs). The majority of personnel are employed as CNS. In total, there are 26.5 WTE for nursing in chronic pain management departments nationally. This amounts to, on average, 0.48 WTE per 100,000 of the population (median = 0.61; range = 0.09–0.73).

There are only 8.03 WTE in total nationally for specialist chronic pain physiotherapy services. This amounts to on average 0.15 WTE per 100,000 of the population per hospital group (median = 0.13; range = 0–0.27). For specialist chronic pain psychology services, there are even less, with only 6.2 WTE for clinical psychology services available nationally for chronic pain management. This amounts to an average 0.1 WTE per 100,000 of the population per hospital group (median = 0.11; range = 0–0.21). Only 12.5% (*n* = 2) of departments have access to dedicated occupational therapy services. No public service in Ireland is currently operating with dedicated clinical pharmacy input.

In terms of services offered, 93.75% (*n* = 15) of chronic pain management departments were offering interventional treatments as part of chronic pain management. In terms of more specialist interventional treatment options, 37.5% (*n* = 6) of departments had the capacity to or were offering advanced neuromodulation therapies (e.g. spinal cord stimulation (SCS)). A percentage of 57.1% of hospital groups (*n* = 4) reported not offering any advanced neuromodulation therapies. Patients were managed in these instances with referral to departments in other hospital groups that do offer these services. 43.75% (*n* = 7) of departments had the capacity to or were offering implantation and management of intrathecal pump (ITP) systems for chronic pain. Geographically, this service is delivered in five of the seven hospital groups (71.4%). An additional two departments who were not providing a service to implant/revise/remove ITP systems offered a refilling service. In this regard, 100% (*n* = 7) of hospital groups were managing ITP systems for chronic pain in some capacity.

There are five Pain Management Programmes (PMPs) being delivered nationally, of which 80% (*n* = 4) have dedicated, protected WTE multidisciplinary staffing. Geographical spread is not homogenous. There are no PMPs being offered by publicly run chronic pain management services in the south and south west parts of the country. There are four self-management programmes (SMPs) being offered nationally, although only 25% of these have dedicated multidisciplinary staffing as described above. 28.6% (*n* = 2) hospital groups are operating neither a PMP nor an SMP.

93.75% (*n* = 15) of departments were accepting referrals for management of chronic pain patients from other hospitals (either within their parent hospital group or outside of their parent hospital group). Seventy-five percent (*n* = 12) of departments were holding regular departmental MDT meetings. Fifty percent of departments (*n* = 8) reported that members of their MDT attended other specialty MDT meetings on a regular basis. The other specialties most commonly cited in this regard were palliative care and oncology.

93.75% (*n* = 15) of departments reported being engaged in clinical audit activities, while 62.5% (*n* = 10) reported having published peer reviewed research in the preceding 12-month period.

**Fig. 1:**
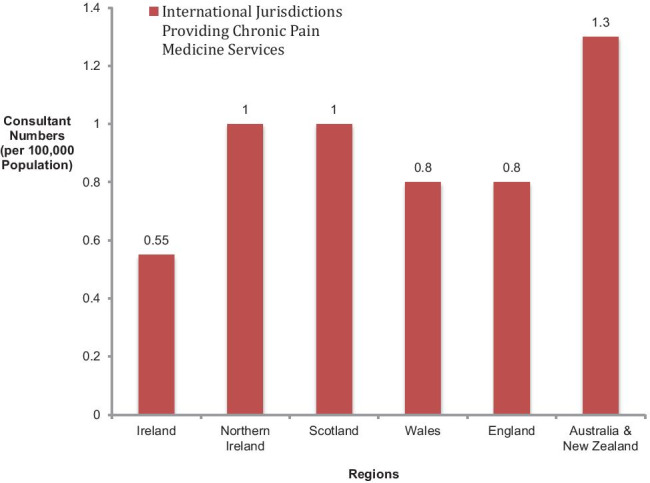
Graphical representation comparing Irish chronic pain management consultant numbers per 100,000 of population to that of international jurisdictions

From a logistical point of view, 100% of departments had dedicated secretarial/administration staff. On average, there was 1.29 WTE of secretarial/administration staff per hospital (median = 1; range = 0.3–3.0); this amounted to an average of 2.95 WTE per hospital group (median = 3; range 1–4.5). A percentage of 62.5% of services (*n* = 10) reported as having a dedicated office space for their department (Fig. 1).

**Fig. 2:**
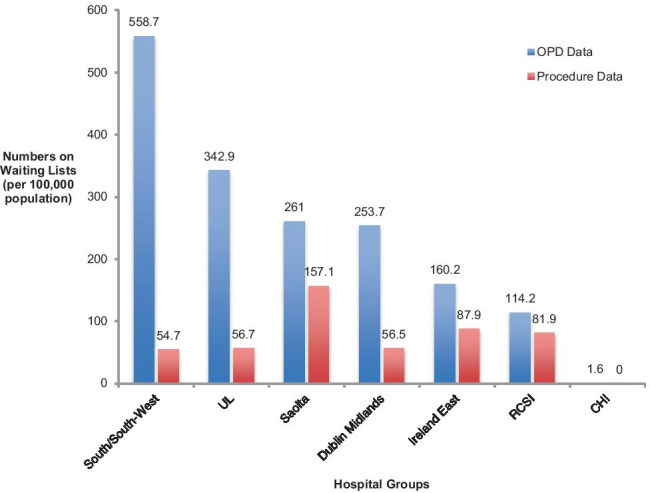
Waiting list data per hospital group expressed as numbers per 100,000 of the population

In terms of waiting lists, data on hospitals were obtained from the national treatment purchase fund (NTPF) website [15]. Waiting list data can be divided into two categories, data regarding those on a waiting list for an outpatient appointment and data regarding those on a waiting list for a procedure. In total (as of January 2020), there were 11,932 patients on the waiting list for chronic pain outpatient clinic appointments; 1912 of these were waiting between 12 and 18 months for an appointment, and 3034 of these were waiting greater than 18 months for an appointment. In terms of those waiting for a procedure (as of January 2020), there were 3496 in total on a waiting list; 403 of these had been waiting between 12 and 18 months while 196 of these had been waiting for more than 18 months. When the data are represented as number of patients per 100,000 of the population, nationally there were 243.6 per 100,000 on an outpatient appointment waiting list; 39 per 100,000 were waiting 12–18 months for an appointment (16%), while 61.9 per 100,000 were waiting > 18 months for an appointment (25.4%). With respect to procedure waiting lists, nationally there were 71.4 per 100,000 of the population on a day case procedure waiting list; 8.2 per 100,000 were on such a waiting list for between 12 and 18 months (11.5%) while 4 per 100,000 were waiting for greater than 18 months (5.6%). Waiting lists by hospital group are shown in Fig. 2.

## Discussion

We undertook a survey of chronic pain management services in all publicly funded hospitals in Ireland in an effort to quantify extent of services and their geographical distribution. As a multidisciplinary delivered medical specialty, reduced staff numbers in comparison to other international jurisdictions is of particular concern in Ireland. With fewer staff available, there are fewer opportunities for patient encounters and this is reflected in the waiting list data.

### The cost of chronic pain

Pain is more than a burden on sufferers and their families alone. The results of the Irish PRIME study (published in 2012) estimated the mean cost per chronic pain patient per annum at €5,665. Extrapolated to total cost per annum of all chronic pain patients in Ireland, this amounted to almost €5.3bn [16]. At the time of publication, this figure would have represented approximately 2.2% of the national GDP (gross domestic product). This would not appear to be a unique phenomenon to Ireland as it is estimated that across the entire EU chronic pain costs Europeans as much as €300bn [17], or around 1.5–3% of GDP [18, 19].

### The cost of delaying healthcare

It is widely accepted that medical care delay or avoidance is likely associated with an increase in morbidity and mortality risk associated with treatable and preventable health conditions [20]. There is a risk that delayed treatment of benign conditions as a result of pandemic‐related cancellations will lead to deterioration in individual patients’ conditions, increasing disability and reducing their ability to work [21]. Perhaps more ominously is the impact on mortality. Severe chronic pain is associated with an increased risk of mortality, independent of socio-demographic factors [22].

### Access to chronic pain management services

According to the International Association for the Study of Pain (IASP) guidelines on wait times, a recommended target maximum waiting time for routine/non-urgent cases is 8 weeks. Other European countries have their own waiting time targets for routine/non-urgent care with Norway striving for 16 weeks, the UK 18 weeks and Finland 6 months [23]. Further afield in Australia, the recommended waiting time target is 6 months [24]. In this context, looking at Irish public waiting list data, the reality is alarming; over 25% of those on waiting lists for public outpatient chronic pain clinic appointments are waiting over 18 months, with 11.5% of those on the waiting list for procedures waiting between 12 and 18 months. Given that these data are from January 2020 and pre-date the COVID-19-related cancellations, this situation most likely has deteriorated.

Long wait times do not appear to be a problem unique to the Irish setting. In the UK, the average waiting times from referral to treatment in chronic pain centres ranges from 6 to 112 weeks. It has been reported that 26% of pain management services in England and Scotland had an average waiting time longer than the 18-week referral-to-treatment target [25]. The results of a recently published study about Canadian services [26] suggest that at least 50% of Canadians with chronic pain are waiting 6 months or more to be seen, and this wait can be as long as 4 years in more rural, isolated Canadian areas. The Waiting-in-Pain II study [24] reported a median wait time for a first appointment for a chronic pain management service in Australia being 60 days. The authors acknowledged however that there remains room for improvement as the range in wait times experienced in many Australian states and territories continued to exceed the recommended maximum wait of 6 months (< 180 days).

### Consultant staffing levels

There are currently 27 consultants providing 16.6 WTEs in publicly funded hospitals for chronic pain management in Ireland. This is largely unchanged in the last 15 years with consultant numbers reported as 28 in 2006 [3] and 28 (with 16 WTEs) in 2017 [6]. International recommendations for staffing levels for chronic pain management suggest a minimum of 1 consultant per 100,000 of population. The need for this in Ireland has been recognised by the College of Anaesthesiologists of Ireland [27] and the HSE’s National Clinical Programme for Anaesthesia [28]. Nationally, our current level of staffing stands at 0.55 consultants per 100,000 of population and 0.34 WTEs per 100,000 of the population. In comparison to our closest neighbours, the UK, this represents significantly fewer specialists. The average number of consultants per 100,000 of the population across the UK is 0.92. Broken down by country within the UK, Scotland and Northern Ireland have the highest levels of consultant staff at 1.0 per 100,000 of the population each, with England and Wales having fewer, both with 0.8 consultants per 100,000 of the population. As is shown in Fig. 1, Ireland is even further behind services in Oceania with combined consultant staffing levels in Australia and New Zealand being reported as 1.3 per 100,000 of the population [29].

### Geographical distribution of services

There is a wide variation in geographical distribution in terms of consultant numbers in Ireland. Hospital group consultant numbers per 100,000 of the population range from 0.34 to 0.73 (This is excluding the data for Children’s Health Ireland (CHI), as this hospital group caters only for the paediatric population of Ireland.) The best staffed hospital group in this regard is the Dublin Midlands Hospital Group with 0.73 chronic pain consultants per 100,000 of the population, delivering care to over 18% of the country’s population. The RCSI hospital group, delivering care to over 22% of the country’s population, has the lowest number of adult chronic pain management consultants per 100,000 of the population at 0.34. Such variations are not unique to the Irish setting [29]. The Royal College of Anaesthetists (UK) Faculty of Pain Medicine acknowledges that at their own national levels cited above, existing resources for pain management services are overstretched [30]. With evidently less consultant resources, the same can be inferred for the delivery of chronic pain management services in Ireland also.

### The multidisciplinary pain clinic in Ireland

IASP recommendations for multidisciplinary pain management centres and clinics are unequivocal in their requirement for services to be considered multidisciplinary. Clinicians who assess and treat patients in the pain management clinic should include physicians, nurses, mental health professionals [7] (e.g. clinical psychologist, psychiatrist), and physiotherapists (physical therapists). The British Pain Society requirements for specialist pain management services go further, stating that services having access to dedicated occupational therapy and clinical pharmacy services is a standard to be adhered to [5]. Seventy-five percent of departments in Ireland reported having dedicated chronic pain nursing staff. Just 31.5% of Irish departments reported having dedicated physiotherapy and psychology input in addition to medical/nursing staff. Data from the 2013 National Pain Audit in the UK showed that 64% and 67% of departments reported having access to dedicated psychology and physiotherapy services, respectively, and 56% of departments having both dedicated physiotherapy and psychology input in addition to dedicated specialist medical staff [4]. Only 12.5% of Irish chronic pain management services had dedicated occupational therapy services. No service reported having access to dedicated clinical pharmacy services. While it seems we are falling short in many areas, there has been some improvement in staffing levels. Since 2017, the total number of chronic pain specialist nursing WTEs has increased from 21.38 to 26.5 [6], while specialist physiotherapy and clinical psychology WTE numbers have both increased from 5.2 and 5.0 to 8.03 and 6.2 WTEs respectively (there is additional funding for one WTE in each discipline; however, these are currently unfilled pending recruitment).

### Multimodal treatments available

In terms of case management, 75% (*n* = 12) of departments operated regular departmental MDT meetings, with 50% (*n* = 8) of departments reporting attending MDT meetings of other services (the most commonly cited departmental MDTs being attended in this regard were palliative care and oncology). The high percentage of departments offering interventional treatments (93.75%, *n* = 15) is largely in keeping with the UK where the rate was 96% in 2013 [9]. Geographically, the availability of advanced neuromodulation therapies (e.g. spinal cord stimulation (SCS)) is not homogenous in Ireland. A significant proportion of the adult population is without direct access to these within their local hospital group, relying on referral to services in other hospital groups for treatment. As an example, the people of the west of Ireland (~ 25% of the national population) have no direct access to a centre providing spinal cord stimulation. Access to intra-thecal pump (ITP) therapies is relatively better, with 43.75% (*n* = 7) of departments (in 71.4% (*n* = 5) of hospital groups) offering them for chronic pain management. Comparison with UK figures published in the national pain audit [9] is difficult, as there is no distinction made between the implant types offered (28% of UK departments were cited as offering implants of any description).

The efficacy of PMPs in improving pain experience, mood, coping, negative outlook on pain and activity levels has been well established [31–33]. PMPs are cost-effective, reduce healthcare consumption and enable more appropriate use of healthcare resources [33, 34]. The BPS has listed PMPs as having level 1 +  + of evidence (high-quality meta-analyses, systematic reviews of randomised controlled trials (RCTs) or RCTs with a very low risk of bias) in this regard [35]. Despite this, only 31.25% (*n* = 5) of departments nationally are offering a dedicated pain management programme. This compares to 61% of departments (as of 2013) in the UK offering the same [4]. Geographical distribution and consequent direct access to services is again an issue in Ireland. With no PMPs being offered by publicly run chronic pain management services in the mid-west, south west and southern parts of the country, 28% of the national population are relying on referral to other hospital groups to access a PMP.

### Organisational structures

BPS core standards for pain management services state that specialised services should have appropriate office space, IT support and administrative staff. With this in mind, we sought to quantify these aspects of services in Irish departments. One hundred percent (*n* = 16) of departments reported having dedicated administrative staff for their service amounting to on average 1.3 WTE per department. Despite this, only 62% (*n* = 10) of departments reported having dedicated departmental office space.

### Study limitations

The strengths of this study include the comprehensive nature of the data collected and the 100% survey response rate. The significant detailed data collected enabled assessment of the state of services around the country in line with international standards and recommendations of best practice. Older national and international data were used for further comparison.

The majority of the data is this study is however based on voluntary reporting from consultant colleagues in hospital settings by invitation. All publicly run chronic pain management services in Ireland were identified as they are known to the authors. This list of services was corroborated with the aforementioned NTPF website database. Most of the questions had binary responses resulting in data that was simple, reliable and unambiguous. Due to the format of the survey, however, it is not possible to verify the completeness or accuracy of the data.

The management of patients with chronic pain in primary care, or community based services, was not surveyed. In the experience of the authors, the delivery of multi-disciplinary care to these patients in the community is rare and not formally coordinated. Therefore, the lack of primary care data is unlikely to underestimate the care available to these patients.

Over 40% of the Irish population have private health insurance, and many of these patients will access private pain management clinics. These clinics and practitioners have not been included in this survey, and consequently this omission does underestimate the overall access to pain management service in Ireland. The majority of these private clinics are however medically operated with a significant emphasis on interventional treatments; they frequently lack significant multidisciplinary input. As per guidance from the core standards for pain management from the BPS, as a specialty we should be moving away from this type of clinic and focus more on multidisciplinary services. Additionally, our government’s vision for the delivery of healthcare in Ireland is to transition to a universal healthcare model with phasing out of private health insurance and early, equitable access to chronic disease management in the community, a policy known as Sláinte Care. We believe our data offers a valuable insight for the Irish Government into the deficits in the system and where resource allocation is required to progress towards the Sláinte Care vision.

## Conclusions

It has been almost 15 years since a statement of need for a national strategy for chronic pain management in Ireland was published [3] and 6 years since the Irish Pain Society appeared before the Oireachtas Committee on Health and Children Debate to propose the development of a National Strategy for Chronic Pain Management. Core to this discussion was prioritizing chronic pain management in healthcare reform [36]. In 2019, the National Clinical Programme for Anaesthesia’s stated a need for an increase in total number of pain management consultant numbers to 44 in order to achieve a level of 1 consultant per 100,000 of population [28]. Three years later, consultant numbers are static at pre-2019 levels.

Ultimately, for many involved in the delivery of chronic pain management services in Ireland, the results of this survey merely confirm what is widely believed and indeed known; a handful of practitioners are delivering a high volume of care within a specialty that is chronically underfunded and under-resourced. A shortage in allied health resources for chronic pain management is of particular concern in Ireland. Patient access to resources is limited by these shortfalls as evidenced by significant waiting lists that exist to access multidisciplinary chronic pain management services throughout the country. The current COVID-19 pandemic restrictions on the delivery of elective and outpatient healthcare are only compounding this issue.

Chronic pain management services in Ireland are very often the end of the referral pathway for many patients. Referrals to pain management services are often from other tertiary care specialities, indicative of the levels of difficulty patients suffering from chronic pain can often experience before getting satisfactory diagnoses, treatments and therapies. In recent years, other specialties with significant numbers of patients with chronic pain (e.g. spinal orthopaedics and rheumatology) have received increased resources to implement multi-disciplinary triage services to address access to care issues. While these services have reduced waiting times, often the end result for these patients is a direct referral to chronic pain management services. Thus, while patients’ waiting times for one service have been reduced by increasing resources, it is simply transferred to another service, with wait times for appropriate chronic pain management therapies and interventions increasing as a consequence of increased referral rates.

While some advances have been made in the delivery of chronic pain management services in Ireland, there remains a significant shortfall that needs to be addressed before they can be considered on equal footing with other international jurisdictions.

## Data Availability

All data and material collected is presented in the manuscript. Clarification on any matter can be made through the corresponding author.
